# Predicting the Risk of Morbidity by GLIM-Based Nutritional Assessment and Body Composition Analysis in Oncologic Abdominal Surgery in the Context of Enhanced Recovery Programs: The PHase Angle Value in Abdominal Surgery (PHAVAS) Study

**DOI:** 10.1245/s10434-024-15143-w

**Published:** 2024-03-23

**Authors:** Marta Sandini, Luca Gianotti, Salvatore Paiella, Davide P. Bernasconi, Linda Roccamatisi, Simone Famularo, Matteo Donadon, Gabriele Di Lucca, Marco Cereda, Edoardo Baccalini, Giovanni Capretti, Gennaro Nappo, Amanda Casirati, Marco Braga, Alessandro Zerbi, Guido Torzilli, Claudio Bassi, Roberto Salvia, Emanuele Cereda, Riccardo Caccialanza

**Affiliations:** 1 Department of Medical, Surgical, and Neurologic Sciences, University of Siena, Siena, Italy; 2 Surgical Oncology Unit, Policlinico Le Scotte, Siena, Italy; 3 School of Medicine and Surgery, University of Milano-Bicocca, Monza, Italy; 4 HPB Unit, Fondazione IRCCS San Gerardo Hospital, Monza, Italy; 5 General and Pancreatic Surgery Unit, Pancreas Institute, University of Verona Hospital, Verona, Italy; 6 School of Medicine and Surgery, Bicocca Bioinformatics Biostatistics and Bioimaging Centre - B4, Milano - Bicocca University, Monza, Italy; 7 Department of Biomedical Sciences, Humanitas University, Pieve Emanuele, Milan, Italy; 8 Department of Hepatobiliary and General Surgery, IRCCS Humanitas Research Hospital, Rozzano, Milan, Italy; 9 Department of Health Sciences, University of Piemonte Orientale, Novara, Italy; 10 Department of Surgery, University Maggiore Hospital della Carità, Novara, Italy; 11 Department of Biomedical Sciences, Humanitas University, Pieve Emanuele, Italy; 12 Pancreatic Surgery, IRCCS Humanitas Research Hospital, Rozzano, Italy; 13 Clinical Nutrition and Dietetics Unit, Fondazione IRCCS Policlinico San Matteo, Pavia, Italy

## Abstract

**Background:**

Preoperative nutritional status and body structure affect short-term prognosis in patients undergoing major oncologic surgery. Bioimpedance vectorial analysis (BIVA) is a reliable tool to assess body composition. Low BIVA-derived phase angle (PA) indicates a decline of cell membrane integrity and function. The aim was to study the association between perioperative PA variations and postoperative morbidity following major oncologic upper-GI surgery.

**Patients and Methods:**

Between 2019 and 2022 we prospectively performed BIVA in patients undergoing surgical resection for pancreatic, hepatic, and gastric malignancies on the day before surgery and on postoperative day (POD) 1. Malnutrition was defined as per the Global Leadership Initiative on Malnutrition criteria. The PA variation (ΔPA) between POD1 and preoperatively was considered as a marker for morbidity. Uni and multivariable logistic regression models were applied.

**Results:**

Overall, 542 patients with a mean age of 64.6 years were analyzed, 279 (51.5%) underwent pancreatic, 201 (37.1%) underwent hepatobiliary, and 62 (11.4%) underwent gastric resections. The prevalence of preoperative malnutrition was 16.6%. The overall morbidity rate was 53.3%, 59% in those with ΔPA < −0.5 versus 46% when ΔPA ≥ −0.5. Age [odds ratio (OR) 1.11; 95% confidence interval (CI) (1.00; 1.22)], pancreatic resections [OR 2.27; 95% CI (1.24; 4.18)], estimated blood loss (OR 1.20; 95% CI (1.03; 1.39)], malnutrition [OR 1.77; 95% CI (1.27; 2.45)], and ΔPA [OR 1.59; 95% CI (1.54; 1.65)] were independently associated with postoperative complications in the multivariate analysis.

**Conclusions:**

Patients with preoperative malnutrition were significantly more likely to develop postoperative morbidity. Moreover, a decrease in PA on POD1 was independently associated with a 13% increase in the absolute risk of complications. Whether proactive interventions may reduce the downward shift of PA and the complication rate need further investigation.

One of the most challenging tasks in major abdominal surgery for cancer is the assessment of the individual risk of having unfavorable postoperative events. Risk assessment might be used to improve patient–physician communication and indeed to allow targeted perioperative optimization of adjustable factors.

Surgery-related morbidity impacts patient recovery, prolongs hospitalization, increases health care costs, causes immunosuppression,^1^ and delays the timing of adjuvant treatments with potential implications on the long-term prognosis in oncologic patients.^2^ Several risk scores, such as frailty scales,^3^ American Society of Anesthesiologists (ASA) classification,^4^ and complexity of the operation^5^ are well acknowledged. Conversely, preoperative nutritional status and body composition are often overlooked or not collected despite potentially playing a role in affecting the short-term prognosis of patients undergoing major surgery.^6^ Moreover, these latter patient characteristics are of increased interest because they are potentially modifiable before surgery.

Although malnutrition is a concern for incremental morbidity, mortality, and costs in the surgical setting, there has been a fundamental lack of consensus on diagnostic criteria. No single existing approach has secured broad global acceptance.^7–10^ In 2019, the Global Leadership Initiative on Malnutrition (GLIM), a core leadership committee with representatives from several clinical nutrition societies, produced new criteria for the diagnosis of malnutrition.^11^ In surgical cancer patients, owing to the limited number of trials assessing postoperative complications and the heterogeneity of the studies, the available data have been considered insufficient to produce valid conclusions on the predictive ability of the new GLIM criteria.^12^

Bioimpedance vectorial analysis (BIVA) is a reliable tool to assess body composition.^13–17^ BIVA works by measuring tissue resistance (R) and reactance (Xc) after alternating current has passed through the body.^18^ The phase angle (PA) is obtained as a ratio between the measures of R and Xc. This angle depends on cellular content and fluids as well as cell membrane integrity and permeability.^17^ As a result, PA is regarded as a marker of cell hydration, vitality, and function.^19^ Thus, altered cellular structure, function, and increased cell death are associated with lower PA values.^20,21^

This study aimed to prospectively evaluate the association of nutritional status, body composition, as measured by BIVA parameters, and the occurrence of surgery-related morbidity.

## Patients and Methods

### Study Overview and Patient Selection

Adult patients scheduled for elective pancreatic, liver, and gastric resection for cancer between June 2019 and September 2022 at three Italian academic medical centers—IRCCS San Gerardo Hospital, Monza, Pancreas Institute, Verona, and Humanitas Research Hospital, Rozzano, Milan—were assessed for inclusion and asked to provide written consent. Exclusion criteria were kidney diseases with glomerular filtration rate < 60 mL/min and presence of compartmentalized fluid collections (pleural effusion or peripheral edema). These conditions may interfere with the electrical property of human tissues, resulting in unreliable body composition variables, such as fat free mass or muscle mass. Further exclusion criteria were: American Society of Anesthesiologists (ASA) score > 3, New York Heart Association class > 2, presence of any infection in the previous 90 days, palliative surgery, and patient refused to sign informed consent. Demographic data, medical history, comorbidity, body mass index (BMI), weight loss, and results from routine blood tests were collected at admission. Malnutrition was defined as per the new GLIM criteria.^11^

The study protocol was approved by the ethical committees of all institutions (no. 0005228).

### Bioelectrical Impedance Assessment

A single-frequency phase-sensitive impedance analyzer (Nutrilab^®^, Akern SRL, Pisa, Italy) was used for BIVA assessments. BIVA was conducted 2 h before the induction of anesthesia (preoperative value) and at postoperative day (POD) 1, 24 h after the end of surgery (postoperative value).

The BIVA method utilizes a phase-sensitive impedance instrument that introduces a constant, low-level alternating current with a tetrapolar surface electrode placement on the hands and feet for whole-body determinations.^22,23^ Impedance (Z) and the delay of current, caused by the lag of current penetrating cell membranes and tissue interfaces, are measured by low Z electrodes, and expressed as PA. Impedance is a complex number that comprises the resistance or purely resistive component (water and electrolytes in fluids and tissues) and the reactance or capacitive component in tissues (cells and tissue interfaces). Complex electronic circuitry permits the determination of the time delay between voltage and current at the cell membrane and tissue level and thus determines the PA. The complex Z of an organism can be differentiated into R and Xc components with simple mathematics; corresponding to Z (sin phase angle) and Z (cos phase angle), respectively, of a R–Xc series circuit for the body. Routinely, a 50 kHz phase-sensitive BIVA instrument measures PA and Z, and calculates R and Xc.

The standardized PA (SPA) is the observed PA–mean phase angle/standard deviation (SD), where the mean and SD are from sex stratified, age stratified, and BMI stratified phase angle reference values. Hydration assessment of patients was conducted through the software Bodygram® (Akern SRL—Pisa, Italy). Details of BIVA principles, measurement methods and definitions have been previously described.^16^ All the other BIVA parameters were calculated using specific equations.^5,24,25^

### Study Endpoints

The primary endpoint of the study was the association of malnutrition and BIVA-derived PA with the occurrence of postoperative complications. The secondary endpoint was the association of all other BIVA estimates with the occurrence of postoperative morbidity.

### Perioperative Care and Outcome Measures

Perioperative care was provided per the Enhanced Recovery After Surgery (ERAS) recommendations.^24–26^ In particular, all patients were allowed to resume oral food “at will” from postoperative day 2 with progressively increasing quantities. Malnourished patients had a supportive enteral nutrition through a nasoenteric feeding tube until they could eat normally. Intraoperative fluid administration with balanced solution was tailored to each patient according to either the variation of the cardiac output or of the pulse pressure variation, through continuous radial arterial monitoring, according to a goal-directed fluid therapy approach. During liver resection, fluid infusion was restricted to obtain a central venous pressure of less than 5 mmHg (through noninvasive estimation of stroke volume variation). Fluids were then restored at the end of the parenchymal transection.

A complication was defined as any deviation from a normal postoperative course and needing any sort of intervention. Morbidity was collected and graded according to the Clavien–Dindo classification (CDC).^27^ The actual duration of hospital stay was also recorded. A 30-day follow-up after discharge of patients for occurrence of complications was performed through office visits. Mortality rate was assessed at 90 days after surgery by telephone interviews.

### Statistical Analysis

Continuous variables are summarized using median and interquartile range (IQR), while categorical variables are reported as numbers and percentages. Patient and operative characteristics were described in the whole cohort and compared between patients with or without complications using the Mann–Whitney *U* test for continuous variables and chi-square test for categorical variables. Preoperative and postoperative BIVA-derived variables were compared within each patient using the Wilcoxon signed-rank test. The absolute delta (postoperative–preoperative) of BIVA-derived parameters was compared in patients with and without complications using the Mann–Whitney *U* test.

The absolute PA variation, defined as the difference between values at POD 1 and preoperative (ΔPA), was considered a potential prognostic marker for postoperative morbidity. The marker was dichotomized according to a priori selected threshold (− 0.5). It was postulated that a 10% reduction of a recognized risk cutoff of 5°^28^ could identify patients with significant loss of cell function and consequently having low functional reserve. The optimal cutoff for PA was determined using receiver operating characteristic (ROC) curve methodology, specifically with the criterion of the maximum Youden index and considering the occurrence of complications as the binary outcome. Variable and multivariable logistic regression models were applied to assess the association between ΔPA and other known prognostic factors with both overall and major postoperative morbidity (patients were considered as clustered within centers). A sample size of 520 patients was fixed to reach 80% power to detect an absolute difference of 12% in the overall risk of postoperative complications between the two ΔPA-defined groups using a two-sided *Z* test with a type *I* error of 0.05.

## Results

During the study period, 542 patients with a median age of 66 years (IQR 57–74 years) were prospectively analyzed. In total, 303 (55.9%) were males and 279 (51.5%) underwent pancreatic resections, 201 (37.1%) underwent hepatobiliary resections, and 62 (11.4%) underwent gastric resections. The prevalence of preoperative malnutrition was 16.6% (90/542).

The overall number of complicated patients was 289 (53.3%), and 84 (15.5%) experienced complications with a CDC grading ≥ 3. When patients with complications were compared with patients without morbidity, the two groups were significantly different for the target organ of surgery, weight loss, ASA score, neoadjuvant treatments, use of laparoscopy, duration of operation, blood loss, use of blood transfusion, and postoperative admission to intensive care units. There was also a center effect on complications (Table 1).

**Table 1:** Descriptive statistics of the whole cohort and comparison of the characteristics of patients with or without complications

Variables	Overall *N* = 542	No complications *N* = 253	With complications *N* = 289	*p*-value*
Center (%)				0.020
1	244 (45.0)	112 (44.3)	132 (45.7)	
2	175 (32.3)	71 (28.1)	104 (36.0)	
3	123 (22.7)	70 (27.7)	53 (18.3)	
Age, years	66.0 [57.0, 74.0]	66.0 [54.0, 73.0]	66.0 [58.0, 74.0]	0.326
Sex				0.854
Male	303 (55.9)	143 (56.5)	160 (55.4)	
Female	239 (44.1)	110 (43.5)	129 (44.6)	
Weight, kg	69.0 [60.0, 80.0]	69.0 [60.0, 80.0]	70.0 [60.0, 79.0]	0.920
Height, cm	167.0 [160.0, 174.0]	168.0 [162.0, 174.0]	167.00 [160.0, 174.0]	0.320
Body mass index	24.4 [22.2, 27.5]	24.0 [21.9, 27.6]	24.6 [22.3, 27.5]	0.362
Weight loss	0.0 [0.0, 4.0]	0.0 [0.0, 2.0]	0.0 [0.0, 5.0]	0.005
Malnutrition (GLIM criteria)	90 (16.6)	34 (13.4)	56 (19.4)	0.082
Site of the neoplasm				< 0.001
Pancreas	279 (51.5)	107 (42.3)	172 (61.9)	
Liver	201 (37.1)	113 (44.6)	88 (30.4)	
Stomach	62 (11.4)	33 (13.0)	29 (10.0)	
Neoadjuvant treatments	113 (20.9)	66 (26.1)	47 (16.3)	0.007
Comorbidities				
Cardiac	92 (17.0)	34 (13.4)	58 (20.1)	0.051
Hypertension	230 (42.6)	107 (42.3)	123 (42.9)	0.964
Diabetes	107 (19.8)	43 (17.0)	64 (22.3)	0.151
Renal	15 (2.8)	4 ( 1.6)	11 ( 3.8)	0.187
Pulmonary	13 (2.4)	9 ( 3.6)	4 ( 1.4)	0.173
Gastrointestinal	91 (16.8)	47 (18.6)	44 (15.3)	0.364
Drug abuse	10 (1.9)	6 ( 2.4)	4 ( 1.4)	0.594
Neurologic	33 (6.1)	12 ( 4.7)	21 ( 7.3)	0.291
Previous cancer	159 (30.1)	86 (35.2)	73 (25.6)	0.021
Other	347 (64.7)	159 (63.6)	188 (65.7)	0.671
Number of comorbidities	2.0 [1.0, 3.0]	2.0 [1.0, 3.0]	2.0 [1.0, 3.0]	0.655
Number of comorbidities				0.383
0–1	165 (30.4)	77 (30.4)	88 (30.4)	
2–3	272 (50.2)	133 (52.6)	139 (48.1)	
4–7	105 (19.4)	43 (17.0)	62 (21.5)	
ASA score				0.001
1	56 (10.4)	39 (15.5)	17 ( 5.9)	
2	328 (60.7)	142 (56.3)	186 (64.6)	
3	156 (28.9)	71 (28.2)	85 (29.5)	
Epidural analgesia	117 (21.8)	53 (21.0)	64 (22.5)	0.752
Type of surgery				< 0.001
Pancreatoduodenectomy	174 (32.1)	58 (22.9)	116 (40.1)	
Distal pancreatectomy	70 (12.9)	35 (13.8)	40 (13.8)	
Total pancreatectomy	25 (4.6)	14 (5.5)	19 (6.6)	
Total gastrectomy	28 (5.2)	15 (5.9)	13 (5.0)	
Partial gastrectomy	34 (6.7)	18 (7.1)	16 (5.5)	
Major hepatectomy	50 (9.2)	20 (7.9)	34 (11.7)	
Minor hepatectomy	151 (27.8)	90 (35.6)	54 (18.7)	
Minimally invasive approach	79 (14.6)	49 (19.4)	30 (10.4)	0.005
Operation time, minutes	360.0 [270.0, 440.0]	328.5 [245.5, 420.0]	380.0 [300.0, 455.0]	< 0.001
Estimated blood loss, mL	300.0 [150.0, 400.0]	200.0 [100.0, 350.0]	300.0 [200.0, 500.0]	< 0.001
Transfusions	57 (10.5)	13 (5.5)	44 (15.8)	< 0.001
Clavien–Dindo grading (%)				
1		NA	68 (12.5)	
2		NA	137 (25.3)	
3A		NA	35 (6.5)	
3B		NA	15 (2.8)	
4A		NA	17 (3.1)	
4B		NA	6 (1.1)	
5		NA	11 (2.0)	
Severe complications (CD ≥ 3) (%)		NA	84 (15.5)	
Intensive care unit (%)	187 (35.0)	55 (22.3)	132 (45.8)	< 0.001
Planned	143 (76.5)	48 (87.3)	95 (72.0)	
For complications	37 (19.8)	NA	37 (28.0)	
Unknown	7 (3.7)	7 (12.7)	0 (0)	

*Mann–Whitney *U* test for continuous variables, chi-square test for categorical variables

Values are median [IQR] or number (%)

*NA* not applicable

All BIVA parameters were significantly affected by the surgical trauma (Table 2). In particular, at POD1, there was a drop in PA, SPA, adipose component, and body cell mass, while total, extracellular, and intracellular water increased with respect to the preoperative values. After dichotomizing the cohort in complicated and noncomplicated patients, the only BIVA parameters that were significantly different were PA, SPA, and body cell mass (Table 3).

**Table 2:** Distribution of BIVA-derived variables in the preoperative and postoperative period

Variables	Preoperative	Postoperative	*p*-value*
Phase angle, degree	5.47 [4.75, 6.20]	4.75 [4.07, 5.50]	< 0.001
Standardized phase angle	− 0.49 [− 1.22, 0.28]	− 1.24 [− 2.06, − 0.48]	< 0.001
Fat free mass, kg	56.42 [49.73, 63.81]	54.42 [47.22, 62.18]	< 0.001
Fat mass, kg	14.15 [9.50, 19.66]	12.02 [7.49, 17.81]	< 0.001
Body cell mass, kg	27.48 [22.94, 32.54]	25.77 [21.94, 31.22]	< 0.001
Skeletal muscle mass, kg	25.98 [21.73, 31.22]	24.67 [20.15, 30.06]	< 0.001
Appendicular skeletal muscle mass, kg	19.77 [17.04, 23.15]	19.60 [16.67, 22.76]	< 0.001
Total body water, L	39.47 [33.74, 45.43]	40.84 [35.35, 46.59]	< 0.001
Extracellular body water, L	18.13 [15.53, 20.80]	19.39 [16.78, 22.28]	< 0.001
Intracellular body water, L	21.29 [17.72, 24.85]	21.49 [18.12, 24.93]	< 0.001
Hydration index	73.63 [73.30, 73.94]	74.50 [73.65, 80.48]	< 0.001
Skeletal muscle mass index	9.23 [8.06, 10.70]	8.75 [7.53, 10.19]	< 0.001
Appendicular skeletal muscle mass index	7.09 [6.27, 7.88]	6.96 [6.19, 7.78]	< 0.001
Fat mass index	5.22 [3.44, 6.98]	4.36 [2.72, 6.23]	< 0.001
Fat free mass index	20.05 [18.42, 21.92]	19.34 [17.58, 21.22]	< 0.001

Values are median [IQR]

*Wilcoxon signed rank test

**Table 3:** Comparison of the absolute delta (postoperative–preoperative) of BIVA-derived parameters in patients with and without complications

Variables	Overall *N* = 542	No complications *N* = 253	With complications *N* = 289	*p*-value*
Phase angle, degree	− 0.57 [− 1.21, − 0.15]	− 0.48 [− 0.96, − 0.05]	− 0.75 [− 1.36, − 0.21]	< 0.001
Standardized phase angle	− 0.64 [− 1.31, − 0.16]	− 0.51 [− 1.05, − 0.06]	− 0.78 [− 1.46, − 0.21]	0.001
Fat free mass, kg	2.21 [− 0.09, 4.54]	2.29 [0.00, 4.62]	2.18 [− 0.34, 4.45]	0.706
Fat mass, kg	− 2.31 [− 4.54, 0.09]	− 2.34 [− 4.62, 0.00]	− 2.09 [− 4.45, 0.34]	0.715
Body cell mass, kg	− 0.86 [− 2.99, 0.71]	− 0.43 [− 2.62, 1.34]	− 1.24 [− 3.49, 0.25]	< 0.001
Skeletal muscle mass, kg	1.59 [− 0.47, 3.51]	1.59 [− 0.31, 3.26]	1.59 [− 0.57, 3.52]	0.823
Appendicular skeletal muscle mass, kg	0.51 [− 0.53, 1.36]	0.52 [− 0.39, 1.47]	0.48 [− 0.57, 1.30]	0.229
Total body water, L	1.79 [− 0.53, 3.94]	1.79 [− 0.35, 3.66]	1.79 [− 0.64, 3.95]	0.823
Extracellular body water, L	1.35 [0.08, 2.81]	1.22 [0.15, 2.71]	1.58 [0.00, 2.94]	0.372
Intracellular body water, L	0.37 [− 0.75, 1.16]	0.44 [− 0.53, 1.26]	0.27 [− 0.88, 1.07]	0.102
Hydration index	0.83 [0.18, 4.73]	0.66 [0.11, 4.22]	0.95 [0.23, 5.14]	0.115
Skeletal muscle mass index	0.58 [− 0.19, 1.29]	0.56 [− 0.12, 1.24]	0.60 [− 0.21, 1.35]	0.976
Appendicular skeletal muscle mass index	0.19 [− 0.18, 0.50]	0.19 [− 0.13, 0.53]	0.16 [− 0.21, 0.46]	0.318
Fat mass index	− 0.79 [− 1.73, 0.03]	− 0.78 [− 1.74, 0.00]	− 0.79 [− 1.71, 0.11]	0.840
Fat free mass index	0.79 [− 0.03, 1.73]	0.78 [0.00, 1.74]	0.79 [− 0.11, 1.71]	0.810

Values are median [IQR]

*Mann–Whitney *U* test

The optimal cut-off of ΔPA, identified using the ROC curve methodology, was − 0.56 which was similar to the a priori chosen cutoff of − 0.5. The sensitivity and specificity at the optimal cutoff were 59.3% and 59.2%, respectively, with a Youden index of 0.185.

The rate of morbidity was 59% in those with ΔPA < −0.5% versus 46% when ΔPA ≥ −0.5 with an absolute difference of 13%, close to what was hypothesized to obtain the sample size (12%). A multivariable logistic regression analysis was performed to evaluate potential pre and intraoperative variables predictive of ΔPA < −0.5 (Table 4). A number of comorbidities ≥ four and diabetes were significantly associated with a risk of having a ΔPA < −0.5, while an ASA score ≤ 2 (OR 0.660; 95% CI 0.415–1.049; *p*-value = 0.079) and a preserved nutritional status (OR 0.602; 95% CI 0.360–1.008; *p*-value = 0.053) appeared somehow protective but without reaching statistical significance.

**Table 4:** Multivariable logistic regression analysis considering as outcome the absolute delta of phase angle < −0.5

Variables	OR (95% CI)	*p*-value
Age, per 10 years	0.972 (0.828;1.142)	0.733
Sex, male versus female	1.257 (0.853;1.852)	0.248
Pancreas versus others	1.344 (0.893;2.022)	0.156
Number comorbidities, 2–3 versus 0–1	0.829 (0.534;1.287)	0.402
Number comorbidities, 4–7 versus 0–1	2.363 (1.206;4.628)	0.012
Diabetes, yes vs no	1.539 (1.322;2.903)	0.019
ASA, 1–2 versus 3	0.660 (0.415;1.049)	0.079
Minimally invasive versus open surgery	1.040 (0.586;1.846)	0.894
Duration of operation, per 60 min	1.050 (0.949;1.161)	0.343
Estimated blood loss, per 100 mL	1.066 (0.988;1.149)	0.097
Malnutrition, no versus yes	0.602 (0.360;1.008)	0.053

*OR* odds ratio, *CI* confidential interval

As exploratory analysis, the predictive value of ΔPA was tested for major complications (CDC ≥ 3). The rate of major morbidity was 18.5% (55/297) in those with ΔPA < −0.5 versus 11.8% (29/245) when ΔPA ≥ −0.5 (absolute difference 6.7%). The *p*-value of the Z test for the difference in proportions was 0.032. The estimated OR (with 95% CI) for ΔPA < −0.5 versus ≥ −0.5 at univariable logistic regression analysis was 1.693 (1.041;2.751) with *p* = 0.034, while at multivariable logistic regression it was 1.677 (0.975;2.884) with *p* = 0.062.

The sensitivity and specificity at the optimal cutoff for ΔPA (− 0.54) for major complications were 50.2% and 65.5%, respectively, with a Youden index of 0.157.

The results of univariable analysis and multivariable logistic regression model for the overall risk of complications are reported in Table 5. Age (OR 1.107; 95% CI 1.003;1.221), pancreatic resections (OR 2.274; 95% CI 1.236;4.183), estimated blood losses (OR 1.199; 95% CI 1.032;1.392), malnutrition (OR 1.767; 95% CI 1.273;2.452), and ΔPA (OR 1.593; 95% CI 1.540;1.647) were significantly and independently associated with the occurrence of postoperative complications in the multivariable analysis.

**Table 5:** Univariable and multivariable analyses for complications

Variables	Univariable		Multivariable	
	OR (95% CI)	*p*-value	OR (95% CI)	*p*-value
Age, per 10 years	1.093 (0.993; 1.205)	0.070	1.103 (0.996; 1.221)	0.060
Sex, male versus female	0.954 (0.752; 1.210)	0.698	0.913 (0.745; 1.120)	0.383
Pancreas versus others	2.356 (1.418; 3.914)	0.001	2.290 (1.131; 4.638)	0.021
Number comorbidities, 2–3 versus 0–1	0.914 (0.574; 1.456)	0.706	0.892 (0.531; 1.497)	0.665
Number comorbidities, 4–7 versus 0–1	1.262 (0.456; 3.494)	0.655	1.275 (0.394; 4.127)	0.685
Diabetes, yes versus no	1.402 (0.807; 2.436)	0.231	1.133 (0.424; 3.028)	0.804
ASA, 3 versus 1–2	1.068 (0.526; 2.171)	0.856	1.128 (0.500; 2.545)	0.772
Duration of operation, per 60 min	1.196 (1.011; 1.414)	0.037	1.054 (0.910; 1.221)	0.485
Estimated blood loss, per 100 mL	1.226 (1.042; 1.443)	0.014	1.200 (1.036; 1.391)	0.015
Malnutrition, yes versus no	1.548 (1.200; 1.997)	0.001	1.807 (1.298; 2.515)	< 0.001
Absolute delta PA, < −0.5 versus ≥ −0.5	1.699 (1.500; 1.925)	< 0.001	1.656 (1.637; 1.647)	< 0.001

For all models, robust standard errors considering centers as clusters are computed

*OR* odds ratio, *CI* confidential interval

## Discussion

The importance of nutritional assessment in oncologic patients undergoing elective major operations is broadly documented.^29^ The present data advocate an independent effect of malnutrition on the occurrence of postoperative morbidity. The pathophysiology of malnutrition in patients with gastrointestinal cancer is multifactorial. From one side, the site of the neoplasm can directly limit food intake owing to symptoms at presentation, such as nausea, loss of appetite, or impaired gastrointestinal outlet. On the other side, the proinflammatory and hormone-like activity of cancer promote and maintain a vicious cycle leading to peripheral insulin resistance, hepatic gluconeogenesis, and protein wasting^30,31^ Despite the acknowledge relationship between cancer and malnutrition, as well as the prognostic value of malnutrition in oncologic surgery, consensus on the diagnostic tools for malnutrition remains controversial, with no actual recommendation on the optimal predictive score in the surgical setting.^32^ In our study we used the newest proposed GLIM criteria.^11^ The novelty of the GLIM malnutrition criteria resides in the combination of etiologic and phenotypic parameters. The assessment of lean mass for the screening of phenotypic criteria should include objective measurements obtained by dual-energy absorptiometry, bioelectrical impedance, ultrasound, computed tomography, or magnetic resonance imaging. In a recent systematic review including 11,700 cancer patients, the prognostic impact of malnutrition on outcomes was analyzed with a meta-analytic approach.^33^ Eight out of ten studies evaluated surgical patients, and in most studies the assessment of fat-free mass was done by measuring calf/arm circumference, or on CT scan. Only one study on esophageal cancer assessed lean mass using bioimpedance. In this study, the diagnosis of malnutrition and severe malnutrition was associated with dismal postoperative and long-term outcomes.^34^ Accordingly, also in our cohort, patients diagnosed with malnutrition as per the GLIM criteria at admission had more than 70% higher risk of developing a complication following major surgery.

More recently, the evaluation of body composition—as added information to the nutritional status—is receiving increasing attention. Among several tools, BIVA represents a safe, minimally invasive, repeatable, usable at bedside, and cost-effective way to measure the body compartments, to assess malnutrition and consequently to stratify the patient prognosis in several oncological, medical, and surgical settings.^28,35,36^ Our study group previously analyzed the utility of repeated BIVA measurement during the perioperative period in predicting the occurrence of severe complications, infections, and pancreatic fistulas following complex pancreatic operations^15,16,37^ and stratifying the risk of postoperative morbidity following liver resections,^5^ However, generalized cutoffs for BIVA parameters, capable of stratifying the short-term prognosis in the oncological surgical setting, have not been provided so far. The main reason may reside in the evaluation of heterogeneous subgroups of cancer patients, with different risk profiles for malnutrition and body compartment alterations, e.g., higher rates of sarcopenia and malnutrition in upper-gastrointestinal (GI) malignancy, compared with higher prevalence of sarcopenic obesity in patients with lower GI cancer.^38,39^ Results from the present study eventually showed that the evaluation of body composition at BIVA can be usefully applied to patients with upper GI cancer for determination of postoperative morbidity risk, and specifically that a decrease in PA at BIVA on POD 1 < −0.5° was independently associated with a 60% increase in the relative risk of overall complications (13% increase in the absolute risk).

It is well-known that during the postoperative period patients experience different level of proinflammatory status, which directly correlates with increased risk of postoperative morbidity^40^ During oxidative stress, reactive oxygen species provoke the disruption of cell membranes, which ultimate leads to a shift of fluid from the intracellular to the extracellular compartment. This produces a modification of the capacitive effect of the membranes, which is one of the main determinants of PA.^18,41^ Compromised cellular structure and health has indeed been associated with lower PA values, and this has been associated with increased inflammatory status in several settings,^42^ thus advising the use of PA as a surrogate for blood inflammatory biomarkers.

Our study suggests that the reduction in PA between pre- and post-surgery may allow to identify those patients with increased inflammatory response, who are more likely to experience an unfavorable postoperative recovery. This attitude to reduced tolerance to stressful events underlies how the PA has been widely associated with the concept of frailty. Not surprisingly, more than four comorbidities and the presence of diabetes mellitus have been identified as independent determinants of the downward shift of PA between pre and postoperatively in our multivariable analysis. The mechanisms as to why some patients are more susceptible to postoperative inflammation may generate different hypotheses. First, not all operations carry the same extent of tissue injury.^40^ Despite including only upper GI malignancies, a heterogeneous variety of procedures have been performed in our cohort. Around one third of the included population underwent a minor hepatectomy, while in 32% of the patients a Whipple operation was performed. Furthermore, given the complexity of the procedure, the rate of postoperative morbidity following pancreatic resection is usually higher when compared with other gastrointestinal operations. Hence, it is not surprising that the multivariable analysis identified pancreatic operations as independent determinants of complication onset, with more than twofold increase in morbidity risk. Still, the effect of delta PA between preoperative and POD1 on complication risk remained independent of the type of operation at the multivariable analysis. This suggests that added information from the BIVA must be recognized. Second, differences according to the perioperative management can be advocated. In the last decades, the role of multimodal strategies—mainly recommended in ERAS programs—has been set. The ERAS programs have been conceived to minimize the surgical insult, reduce the extent of postoperative inflammation, and subsequently lower the risk of complications.^43^ All patients in our study were managed according to ERAS principles. Specifically, the intraoperative management of intravenous fluid was standardized at all participating centers, thus reducing the risk of fluid overload in the extracellular compartment. The goal of maintaining normal hydration during the surgical procedure reduced the magnitude of PA drop on POD1, as previous clinical studies clearly showed a downward shift of the PA vector as consequence of overhydration.^44^

The independent effect at the multivariable analysis of malnutrition and PA must be underlined. Our data not only further validated the use of BIVA for screening and classification of malnutrition according to the GLIM recommendation, but also provided additional information beyond impaired nutritional status alone. A recent review focused on the role of SPA at BIVA to assess nutritional status in cancer. SPA correlated with several biometrics of muscle mass and functions.^45^ The presence of malnutrition and low PA remained independent determinants of unfavorable postoperative recovery. Impaired cell vitality and function significantly correlate with a reduction in the patient’s capability to tolerate stress conditions,^17,46^ such as surgical trauma. In our oncological surgical cohort, baseline BIVA values were mostly in the normality range. However, the repeated BIVA on POD1 identified a subgroup of subjects, in whom the initiated cellular derangement produced a significant drop in the PA and increased the risk of complications. Hence, we hypothesize that this preclinical warning may be unveiled through the assessment of delta PA between the pre and postoperative phases, and may represent an earlier and independent surrogate for patient frailty. Those patients appear to be at increased risk of postoperative morbidity and should be adequately observed to achieve prompt diagnosis and interventions.

Some limitations of this study should be disclosed. First, we were unable to establish a unique PA cutoff determining the risk of postoperative complications. Rather, we observed that a high downward shift over time was significantly associated with patient morbidity. However, our results could allow a better generalization of BIVA in surgical settings, as the shift over time remains independent of the absolute values and their determinants. Second, despite showing a trend, we were unable to confirm a predictive ability of ΔPA on the occurrence of major morbidity. However, we calculated the sample size on the estimated rate of overall complications, and we observed a 15.5% rate of severe complications. The lack of statistical significance can possibly be attributed to a relatively small sample size and consequent beta error. Moreover, even though all patients were perioperatively managed according to ERAS procedures, compliance to the protocol was not assessed. It has been shown that the compliance with ERAS items among centers, when assessed individually for each patient, can vary widely, and is seldom higher than 30%, depending on the type of operation.^47^ Thus, the rate of compliance may represent a confounder. However, for all multivariable models, robust standard errors, considering centers as clusters, have been computed.

Moreover, the application of our ΔPA should be further investigated and validated with different bioimpedance analyzers. Last, potential beneficial effects of preoperative active interventions have not yet been explored. Although the catabolic effect of cancer is firmly connected with an increased systemic inflammatory response and is not responsive to standard nutritional interventions,^9^ the perioperative use of specific nutrition supplements—namely immunonutrition—demonstrated a significant effect in improving surgical outcomes.^48^ More recently, multidimensional prehabilitation programs have shown clear benefits on postoperative morbidity risk.^49^ Potential effects of immunonutrition and prehabilitation on PA in an ERAS setting may represent a future field of research.

In conclusion, preoperative nutritional assessment and evaluation of delta PA at BIVA may help identify patients at risk of morbidity following major oncologic abdominal surgery, who may benefit from strict monitoring to anticipate diagnosis and provide prompt treatment. Whether proactive interventions may modify the response to surgical stress, and consequently reduce the downward shift of PA and the complication rate remains to be further investigated.
